# Is-there a place for vagus nerve stimulation in inflammatory bowel diseases?

**DOI:** 10.1186/s42234-018-0004-9

**Published:** 2018-04-03

**Authors:** Bruno Bonaz

**Affiliations:** 1 Division of Hepato-Gastroenterology, University Hospital, Alpes, F-38000 Grenoble, France; 2 University Grenoble Alpes, Grenoble Institute of Neurosciences, GIN, Inserm U1216, F-38000 Grenoble, France; 3 Division of Hepato-Gastroenterology, CHU Grenoble Alpes, -10217, 38043 Grenoble Cedex 09, CS France

**Keywords:** Vagus nerve, Vagus nerve stimulation, Inflammatory bowel diseases, Cholinergic anti-inflammatory pathway

## Abstract

The vagus nerve (VN), the longest nerve of the organism that innervates the gastrointestinal tract, is a mixed nerve composed of 80% of afferent and 20% of efferent fibers. The VN has anti-inflammatory properties, in particular an anti-TNFα effect through the cholinergic anti-inflammatory pathway. The VN is a key component of the autonomic nervous system, i.e. the parasympathetic nervous system. An imbalance of the autonomic nervous system, as represented by a low vagal tone, is described in many diseases and has a pro-inflammatory role. Inflammatory bowel diseases (IBD) are chronic disorders of the gastro-intestinal tract where TNFα is a key cytokine. VN stimulation (VNS), classically used for the treatment of drug resistant epilepsy and depression, would be of interest in the treatment of IBD. We have recently reported in a 6 month follow-up pilot study that VNS improves active Crohn’s disease. Preliminary data of another pilot study confirm this interest. Similarly, VNS has recently been reported to improve rheumatoid arthritis, another TNFα mediated disease. Bioelectronic Medicine, as represented by VNS, opens new therapeutic avenues in the treatment of such chronic inflammatory disorders. In the present manuscript, we will focus on the interest of VNS in IBD.

## Background

Inflammatory bowel diseases (IBD) are chronic inflammatory disorders of the gastrointestinal tract involving the recto-colon for ulcerative colitis (UC) and all the digestive tract (essentially the ileum and/or colon) for Crohn’s disease (CD) (Abraham & Cho, 2009). Flares are characterized by abdominal pain, diarrhea (bloody in UC), fever, weight loss, and extra-intestinal manifestations (skin, eyes, joints) with a significant impact on quality of life. About 2.2 million people in Europe and 1.5 million Americans are affected by IBD (Molodecky et al., 2012) with a regular increase of their incidence and prevalence due to the “Westernization” of our lifestyle. The pathophysiology of IBD involves genetic, immunologic and environmental factors (de Souza, 2017). The concept of medical treatment of IBD has recently evolved from steroids/immunosuppressants to biologics targeting TNFα, a key cytokine in IBD, and more recently anti-adhesion molecules and anti-IL12/23, with the aim to heal mucosa and to prevent irreversible damages of the digestive tract (Chang & Hanauer, 2017). However, these treatments don’t cure the disease and are not fully effective because patients are either non-primary responders or secondarily lose response (Ben-Horin et al., 2014). In addition, these drugs are not devoid of side-effects (Bonovas et al., 2016) thus explaining that patients are reluctant and not compliant with these treatments (Lenti & Selinger, 2017), and are among the highest users of complementary and alternative medicines (Yanai et al., 2016).

Consequently, a non-drug therapy targeting an anti-inflammatory pathway and devoid of side-effects would be of interest in the treatment of IBD. Bioelectronic Medicine is a new therapeutic approach using devices to modulate electrical activity of the nervous system to restore organ function and health without the side-effects of pharmaceutical agents and avoiding compliance problems (Olofsson & Tracey, 2017). Among the nervous system, the vagus nerve (VN), the principal component of the parasympathetic nervous system, appears as an interesting target for Bioelectronic Medicine due to its anatomical specificity at the interface of gut-brain interactions and its role in the neuro-endocrine-immune axis (Bonaz et al., 2017). VN stimulation (VNS) is presently used in the treatment of drug resistant epilepsy and depression (Milby et al., 2008) based on the widespread central projections of the VN (Ruffoli et al., 2011). More recently, VNS has been shown to improve experimental models of septic shock and colitis (Borovikova et al., 2000; Meregnani et al., 2011). Translational pilot studies have been recently performed in chronic inflammatory disorders such as CD and rheumatoid arthritis (Bonaz et al., 2016; Koopman et al., 2016). In the present review, we will focus our attention on the use of VNS in IBD, especially in CD.

## Rationale for targeting the vagus nerve in inflammatory bowel diseases

**Fig. 1:**
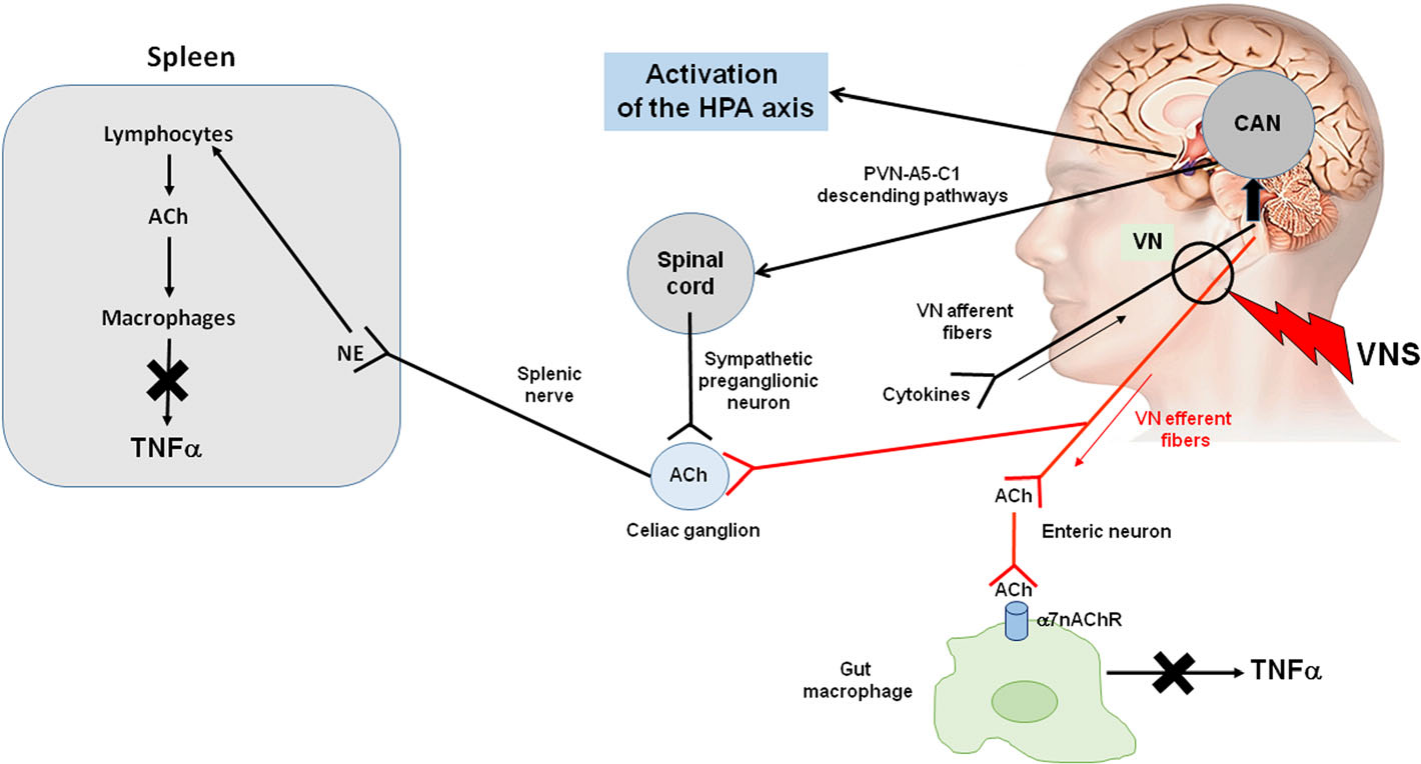
Anti-inflammatory pathways of the vagus nerve. Adapted from reference (Bonaz et al., 2017). Ach, acetylcholine; CAN, central autonomic network; HPA axis, hypothalamic pituitary adrenal axis; NE, norepinephrine; TNF α, tumor necrosis alpha; VN, vagus nerve; VNS, vagus nerve stimulation; α7nAChR, alpha7 nicotinic acetylcholine receptor

The VN is the longest nerve of the organism innervating all the digestive tract for some anatomists (Delmas & Laux, 1933). It is a mixed nerve composed of 80% afferent and 20% efferent fibers (Prechtl & Powley, 1990). The VN is a key element of brain-gut interactions and of the autonomic nervous system (Bonaz et al., 2017). There is an imbalance of the autonomic nervous system in IBD that could play a role in the pathophysiology of IBD. Indeed, we have reported that vagal tone was significantly blunted in IBD in relation with negative affects and a high TNFα level (Pellissier et al., 2010; Pellissier et al., 2014). The VN, through its afferent fibers, activates the hypothalamic-pituitary adrenal (HPA) axis (Fig. 1) known to dampen peripheral inflammation through the release of glucocorticoids (Harris, 1950). Another anti-inflammatory pathway, involving vagal efferents, has been more recently described in 2000 by the group of Tracey and called the cholinergic anti-inflammatory pathway (CAP) (Pavlov et al., 2003). Indeed, the release of acetylcholine (ACh) at the distal end of the VN is able to inhibit the release of TNFα by macrophages through the link of ACh with alpha7nicotinic receptors (α7nAChR) of these macrophages (Wang et al., 2003) (Fig. 1). However, the VN does not innervate directly resident macrophages in the gut but through an interaction with nNOS-VIP-Ach interneurons with their nerve endings in close proximity of these resident macrophages (Cailotto et al., 2014). The group of Tracey has also described a vago-sympathetic pathway where, in a synergistic effect, the VN interacts with the sympathetic splenic nerve releasing norepinephrine acting on β2 receptors of splenic CD4 T-lymphocytes which release ACh to inhibit the release of TNFα by splenic macrophages through an interaction with their α7nAChR (Rosas-Ballina et al., 2008) (Fig. 1). For some authors the sympathetic nervous system, through the release of its neurotransmitter, norepinephrine, could be the efferent arm of the CAP (Martelli et al., 2016). Indeed, vagal afferents activate the central autonomic network (Benarroch, 1993) which in return, through descending pathways from the paraventricular nucleus of the hypothalamus, the A5 noradrenergic group and the C1 adrenergic group, modulates pre-ganglionic neurons of the sympathetic nervous system in the spinal cord and thus the splenic nerve (Abe et al., 2017) (Fig. 1). Consequently, the interaction of the VN with the sympathetic nervous system is of interest and targeting the anti-inflammatory effect of the VN, i.e. an anti-TNFα pathway, both through its afferent and efferent fibers would be of interest in IBD.

## How to target the vagus nerve?

Based on its dual anti-inflammatory role, the VN appears as an interesting therapeutic target for chronic inflammatory disorders such as IBD, in particular targeting the CAP. This approach can be obtained *i)* pharmacologically with α7nAChR agonists such as nicotine (Cui & Li, 2010), GTS21 (Kox et al., 2011), and AR-R17779 (van Westerloo et al., 2006), activation of the central cholinergic pathway with an AChesterase inhibitor (galantamine) (Pavlov et al., 2009) or CNI-1492, a cytokine inhibitor and synthetic guanylhydrazone mitogen-activated protein kinase blocker (Tracey, 1998), *ii)* high fat enteral feeding inducing the release of CCK that activates CCK1 vagal afferents thus activating the CAP (Luyer et al., 2005), *iii)* complementary and alternative medicines such as meditation, yoga, acupuncture, hypnosis, known to activate the VN (Keefer et al., 2013), i*v)* physical exercise (Mora et al., 2007), *v)* VNS which appears as the most interesting tool because already validated in drug resistant epilepsy and depression in human with very few side effects (Bonaz et al., 2013).

## Vagus nerve stimulation in epilepsy and depression

The first VNS device for the treatment of drug-resistant epilepsy was introduced in human in 1990. VNS has been validated by the US Food and Drug Administration (FDA) for drug resistant epilepsy in 1997 and in 1994 for Europe and for depression in 2005. For these indications, the mechanism relies on an activation of vagal afferents by high frequency stimulation at 20–30 Hz (Bonaz et al., 2013). Indeed, low frequency stimulation is less efficient in epilepsy than high frequency stimulation and stimulation over 50 Hz induces damage of the VN (Schachter & Boon, 2007). Brain imaging studies in human have shown that brain regions activated were greater at high (20 Hz) than low (5 Hz) frequency VNS (Lomarev et al., 2002). The widespread projections of the NTS, the target of vagal afferents, in the brain explain the efficacy of VNS in epilepsy and depression (Ruffoli et al., 2011). The locus coeruleus (LC), the principal brain noradrenergic nucleus, is believed to mediate many of the effects of VNS in the central nervous system. The LC receives excitatory input from the NTS via the nucleus paragigantocellularis (Ruffoli et al., 2011). Lesions of the LC block the anti-epileptic and anti-depressant effects of VNS (Krahl et al., 1998). VNS increases firing rates of LC neurons and norepinephrine concentrations in the cortex and hippocampus, two projection sites of the LC (Dorr & Debonnel, 2006; Roosevelt et al., 2006) and activates the LC in a fMRI study in humans (Frangos et al., 2015). VNS may act initially and/or predominantly on the LC, and indirectly with the dorsal raphe nucleus via afferents from the LC (Dorr & Debonnel, 2006).

A reduction of seizure is observed in 50% of patients after 2 to 3 years of VNS (Morris 3rd & Mueller, 1999). In a retrospective study of VNS in 65 patients with epilepsy with a mean duration of VNS of 10 years Elliott RE et al. (Elliott et al., 2011) showed that the mean reduction in seizures at 6 months and years 1, 2, 4, 6, 8, and 10 was 35.7, 52.1, 58.3, 60.4, 65.7, 75.5, and 75.5%, respectively. Consequently, VNS is a slow acting therapy.

## Vagus nerve stimulation in inflammatory bowel diseases

Based on its use in epilepsy and depression, VNS could be an interesting tool for the treatment of IBD based on pre-clinical data in rats with colitis and 2 recent clinical pilot studies targeting 2 different populations of patients with active CD either naïve of anti-TNF on inclusion or in patients resistant to biologics.

### Experimental rationale

The autonomic nervous system, in particular the parasympathetic nervous system, has a role in the control of experimental colitis. Classically, vagotomy aggravates colitis (Ghia et al., 2006) and sacral nerve stimulation enhances intestinal barrier repair in acute mucosal injury of experimental colitis (Brégeon et al., 2016). The first pharmacological study targeting the CAP used Achesterase (AChE) inhibitors (physostigmine and neostigmine) prior to induce colitis in rats. Physostigmine, which crosses the blood brain barrier, induced a greater improvement of colitis than neostigmine pretreatment thus suggesting than central cholinergic pathways have a greater protective effect than peripheral pathways (Miceli & Jacobson, 2003). Based on this study and the description of the CAP, our group has performed low frequency (5 Hz) VNS in chronically implanted freely moving rats with the other parameters classically used for epilepsy, supposed to activate vagal efferents, for 5 consecutive days in rats with trinitrobenzenesulfonic acid (TNBS) colitis, classically used as an experimental model of CD (Meregnani et al., 2011). Colonic inflammation was evaluated by a global multivariate index of colitis taking into account *i)* body weight, temperature and locomotor activity, *ii)* areas of lesions and histological damage, *iii)* biological parameters such as myeloperoxidase activity, cytokine and cytokine-related mRNAs at the level of colonic damage and just above. VNS significantly improved the multivariate index of colitis (Meregnani et al., 2011). The inflammatory infiltrate just immediately above the major colonic lesion was significantly improved by VNS while a slight reduction was observed in the lesion thus arguing for a major efficacy of VNS on tissues that are less damaged (Meregnani et al., 2011). Sun et al. (Sun et al., 2013) performed chronic VNS (0.25 mA, 20 Hz, 500 ms) in a model of TNBS colitis in rats and recorded heart rate variability (HRV) as a marker of the sympatho-vagal balance. They observed a significant improvement of colitis under VNS, a decrease of pro-inflammatory cytokines (TNFα and IL-6), and an improvement of HRV. More recently, Jin et al. (Jin et al., 2016) showed that chronic VNS improves inflammation in TNBS-treated rats by inhibiting pro-inflammatory cytokines via an autonomic mechanism; addition of noninvasive electro-acupuncture to VNS enhanced the anti-inflammatory effect of VNS.

### Clinical rationale

In a translational approach, we have performed a pilot study of VNS in CD patients. We have implanted 9 patients with CD, with 8 out of 9 patients with active CD on inclusion. The patient in clinical and endoscopic remission on inclusion had received a treatment with budesonide, a topically acting corticosteroid with extensive first pass hepatic metabolism, and azathioprine, an antimetabolite therapy. This patient stopped budesonide few weeks before inclusion and azathioprine because of a pseudo-flu-like syndrome due to azathioprine. Patients between 18 and 65 years had a Crohn’s disease activity index (CDAI; a research tool used to quantify symptoms of patients with CD such as abdominal pain, number of liquid or soft stools, general well-being) between 220 and 450 (i.e. moderate to severe CD; CDAI< 150: clinical remission), with a small bowel (ileum) and/or colonic CD, diagnosed for more than 3 months, naıve of treatment or despite a stable treatment reference, with a C-reactive protein (CRP; acute-phase protein of hepatic origin) > 5 mg/L and/or a fecal calprotectin (a marker of intestinal inflammation) > 100 μg/g and a Crohn’s disease endoscopic index of severity (CDEIS; for endoscopic assessment of mucosal disease activity. CDEIS< 6: endoscopic remission) ≥7 were prospectively included. Patients under infliximab or any other anti-TNFα agent on inclusion were not eligible. Stimulation parameters were: 10 Hz, 500 μsec pulse width, no more than 1.5 mA, 30 s ON, 5 min OFF. Intensity was progressively increased during the first month after implantation from 0.25 to 1.50 mA. All patients signed an informed consent. The study was approved by the institutional ethics committee (11-CHUG-28) and registered in ClinicalTrials.gov (Identifier: NCT01569503; First received: March 30, 2012). The primary endpoint was to induce clinical remission (CDAI< 150) and the secondary endpoints to induce biological and endoscopic remission and restore vagal tone. The first patient was implanted on April 2012 and the last one on March 2016. All the patients entered in a one year follow-up study. Only two patients were under azathioprine (2.5 mg/kg) on inclusion. VNS induced a deep (clinical-biological-endoscopical) remission in 5 of the nine patients with restored vagal tone. The patient on remission on inclusion was still in deep remission under VNS alone at 1 year. The last (9th) included patient has just reached the one year follow-up and is in flare of the disease but did not want to switch to anti-TNF because he was afraid of the side effects of anti-TNF. Two patients were removed from the study after 3 months of VNS despite a clinical improvement during the two first months of VNS and switched to infliximab and azathioprine; one of the two patients was operated (ileocecal resection). These two patients had the highest CDAI, CRP and CDEIS on inclusion as well as the 9th patient included which suggests that VNS, as a slow-acting therapy, is more indicated in moderate CD. All the 9 patients have still the device in place. Among the two patients who left the study at 3 months, the intensity was decreased to 0.5 mA for one patient and stable at 1.5 mA for the other patient. VNS was well tolerated with the classical minor side effect represented essentially by hoarseness. We did not observe any problem of infection either local or systemic and no VNS device was removed. The data on the first seven implanted patients after a 6-month follow-up were recently reported for the first time (Bonaz et al., 2016).

In a second pilot study (SetPoint Medical Corporation, ClinicalTrials.gov Identifier: NCT02311660; First received: December 3, 2014), D’Haens et al. (D’Haens et al., 2016) have reported in an abstract form the effect of VNS for 16 weeks in 8 patients with biologic-refractory small bowel and/or colonic CD. Patients had a moderate to severe CD (220 < CDAI< 450) on inclusion, a CRP ≥ 5 mg/L, a fecal calprotectin ≥200 μg/g, an endoscopic score of activity (SES-CD: Simple Endoscopic Scale for Crohn’s Disease, an endoscopic score correlated with the CDEIS) with the presence of a minimal ulcer score of 2 or 3 in at least 1 segment, with a history of inadequate response and/or intolerance or adverse events to one or more TNF-alpha inhibitors (e.g.,infliximab, adalimumab, or certolizumab pegol), a 8 weeks washed out of biologics. Stimulation parameters were: 10 Hz, 250 μsec pulse, 2 mA max, for 60 s first then for 5 min. At 8 weeks, stimulations were increased if CDAI remission was not achieved. Some of the patients had prior Crohn’s surgery. 8/8 had prior anti-TNF, 4/8 vedolizumab, 2/8 ustekinumab, 8/8 corticosteroids, 5/8 azathioprine, 1/8 mercaptopurine, and 4/8 methotrexate. The primary endpoint was the change in CDAI from baseline to week 16 visit: CDAI scores were reduced by 70 in 6 to 8 patients. The secondary endpoints were *i)* the rate of clinical response at week 16 visit defined as CDAI improvement from baseline of at least 70 points that was obtained in 6/8 patients, *ii)* rate of clinical remission at week 16 visit defined as CDAI≤150: 3/8 patients were in clinical remission, *iii)* change in total SES-CD score from baseline to week 16 visit: endoscopic scores were reduced in 6 to 8. CRP and fecal calprotectin levels were reduced in patients who achieved clinical response. HRV was increased in 6 patients, consistent with an increasing parasympathetic tone. Nine serious adverse events were reported in 5/8 patients, all of which were CD-related except for 1 patient with a device-related postoperative infection.

These two pilot studies show a sign in favor of an effect of VNS in active CD and are complementary since our study involved patient naïve of biologics on inclusion and with only 2 patients under azathioprine on inclusion while in the study of D’Haens et al. (D’Haens et al., 2016) highly refractory patients were included with failure of biologics. Of course a more robust randomized control trial needs to be performed, in particular including patients who are naïve of or refractory to biologics.

Very recently, Koopman et al. (Koopman et al., 2016) have shown that VNS was able to inhibit peripheral blood production of cytokines (TNFα, IL-1β, and IL-6) and attenuate disease severity in rheumatoid arthritis, another TNF-mediated disease.

## Noninvasive or invasive VNS?

Classically VNS performed in epilepsy and depression as well as in the 2 pilot studies in CD patients is invasive, generally performed by a neurosurgeon familiar with the technique with a duration of 1 h, with few side-effects. However, some patients are reluctant to surgery a fortiori in a vasculo-nervous region involving the vein and the external carotid artery which are in close contact with the VN, thus noninvasive (n) VNS would be valuable. In addition, if the device can be removed, the electrode wrapped around the VN is generally left in place although some authors removed it without major damage to the nerve and vessels (Champeaux et al., 2017). Devices stimulating the VN at the cervical level or at the auricular level have been developed. Indeed, the cymba concha of the external ear is innervated by a sensory auricular branch of the VN (Peuker & Filler, 2002) that sends projection in the NTS in cats (Nomura & Mizuno, 1984) and humans (Frangos et al., 2015). Transauricular (ta) VNS could thus activate the CAP through an inflammatory reflex. ta-VNS dampened LPS-induced inflammatory responses in rats that was suppressed by vagotomy or α7nAChR antagonist (Zhao et al., 2012). External stimulation of the left cervical VN decreased whole blood culture-derived cytokines and chemokines in healthy volunteers (Lerman et al., 2016). Two n-VNS devices, used for epilepsy, depression, and headache are available, the NEMOS one (Cerbomed, Erlangen, Germany) using an intra-auricular electrode (Stefan et al., 2012) and the GammaCore one (electroCore LLC, Basking Ridge, NJ, USA) with two stainless steel round discs functioning as skin contact surfaces (Nesbitt et al., 2015). There are presently no published data regarding the use of these two devices in inflammatory disorders of the digestive tract.

No significant serious adverse events have been reported with these noninvasive devices. By comparison to invasive VNS, n-VNS has the disadvantage of its compliance which is an important problem in the treatment of IBD. Indeed, about 30–40% of IBD patients don’t take their treatment (Herman & Kane, 2015). In addition, complementary and alternative medicine-IBD users are less likely to be adherent to medical therapy than nonusers (Nguyen et al., 2016). One can wonder if it could be the same problem with these noninvasive devices. In addition, in the case of the Gammacore device, the reproducibility of the position of the discs in contact with the VN is questionable. Finally, in an experimental model of septic shock, ta-VNS was less efficient than VNS to attenuate the LPS-induced serum cytokine (TNF*α*, IL1*β*, and IL6) response (Zhao et al., 2012).

## Questions-future for vagus nerve stimulation in inflammatory bowel diseases

The use of VNS in IBD, wether invasive or noninvasive, needs convincing data for health-authorities for regulatory approval and reimbursement as well as for the community of IBD physician, and patients who are expecting a nondrug therapy devoid of side-effects. Consequently a robust randomized controlled trial looking at VNS vs sham-stimulated in IBD patients is warranted. Controls should be implanted but not stimulated. Indeed, even low frequency VNS (1 Hz) discretely affected the level of c-fos expression in the rat NTS, compared to sham-operated animals (Osharina et al., 2006). Regarding the frequency of stimulation, if low frequency (5–10 Hz) is supposed to activate vagal efferents (Bonaz et al., 2013), we have reported in rats that even low frequency stimulation at 5 Hz was able to induce modifications of activation in the NTS, the first target of the VN in the brain, as well as in numerous areas of its brain projections (Reyt et al., 2010). Brain imaging studies in human as well as *c-fos* activation in the NTS and other NTS brain related nuclei have been reported in humans and animals under VNS respectively (Lomarev et al., 2002; Osharina et al., 2006). Based on the involvement of both vagal afferents and efferents in the anti-inflammatory effect of the VN one can wonder that VNS at 10 to 30 Hz would be of interest in humans. The intensity of stimulation is generally limited by side-effects such as pain in the throat which generally disappears when decreasing intensity and/or pulse width. Generally intensity beyond 1.50 mA was not well supported in our pilot study in CD patients (Bonaz et al., 2016).

Another question is the duration of the stimulation. Generally, in epilepsy and depression the parameters are 30 s ON and 5 min OFF. This is the timing that we used in our pilot study. However, Koopman et al. (Koopman et al., 2016) performed VNS for 60 s up to 4 times daily in patients with RA based on a previous work where VNS delivered once daily for 60 s attenuated joint swelling, inhibited cytokine production and conferred significant protection against synovitis and periarticular bone erosions (Levine et al., 2014). D’Haens et al. (Krahl et al., 1998) in their preliminary unpublished study also used intermittent VNS (1 to 5 min per day). However, it means that the parameters of the device need to be changed manually with the magnet generally supplied with the device that is not always convenient except if the controller is given to the patient. Consequently, a programmable device would be valuable.

Miniaturization of the VNS device is also warranted. In the same way, instead of an electrode, a VNS device which would act as an electrode by clipping it around the VN would be of interest (see setpointmedical.com; MØ1-ØØ1123). In that case, a single surgical incision would be sufficient thus reducing the duration of the surgery. Another important progress would be a device that is able to record vagal tone and able to trigger VNS in case of low vagal tone to restore a normal tone. A VNS system, AspireSRTM, already approved in Europe, and created by Cyberonics Inc. analyzes relative changes of heart rate, particularly ictal tachycardia, and responds to seizures automatically.

Anti-TNFα drugs are presently the best treatment to prevent postoperative recurrence of CD (Qiu et al., 2015). Surgery cures CD lesions and since VNS is a slow-acting therapy, it could be an interesting tool in such patients. Another possibility would be to use combotherapy (drugs + VNS) to induce mucosal healing with biologics and then VNS could take over the time of its efficacy. The two pilot studies of VNS in IBD (Bonaz et al., 2016; D’Haens et al., 2016) focused on CD but VNS in UC, which involves the recto-colon, would be also of interest. IBD occurs in childhood-adolescence in about 25% (Sawczenko et al., 2001). VNS for epilepsy in children younger than 12 years is off-label but pediatric studies have reported comparable efficacy as for adult patients (Elliott et al., 2011). Consequently, VNS in children with IBD should be of interest.

The cost of VNS was saved within 2 years following implantation of the device in drug resistant epilepsy (Boon et al., 1999). In the same way, the treatment of IBD is estimated to be reduced with VNS. The total modeled per patient infusion therapy costs in year 1 with infliximab was $38,782; drug acquisition cost was the largest total costs driver (90–93%) (Afzali et al., 2017). The cost of the device (neurostimulator + electrode) is ~ $11,000. The battery lasts between 7 and 10 years depending on the frequency of stimulation and intensity of the current. Battery life is correlated with charge, the lower pulse widths (250 and 130 μs) and their respective threshold currents conserve battery life more effectively than the longer pulse widths (500, 750, or 1000 μs) (Helmers et al., 2012). Battery demand is also affected by duty cycle and signal frequency (increases in both of these parameters will negatively affect battery longevity) (Helmers et al., 2012).

VNS is intermittent and regular as classically programmed. There is no special rule to follow in programing of VNS but adjustment to higher settings of the parameters is usually performed progressively, particularly for intensity. High stimulation (30 Hz, 30 s on, 5 min off, 500 μsec pulse width) is more effective than low stimulation (1 Hz, 30 s on, 90–180 min off, 130 μsec pulse width) in epilepsy (Schachter & Boon, 2007). Higher output current is necessary if no improvement is observed in the early phase of VNS in epilepsy; 20% of non-primary responders showed response after an increase of current intensity (Bunch et al., 2007). Consequently, in IBD such an adaptation could be performed.

In a very recent elegant study, Hulsey et al. (Hulsey et al., 2017) recorded neural activity in the LC in response to VNS over a broad range of current amplitudes, pulse frequencies, train durations, inter-train intervals, and pulse widths. Brief 0.5 s trains of VNS drive rapid, phasic firing of LC neurons at 0.1 mA. Higher current intensities and longer pulse widths drive greater increases in LC firing rate. Varying the pulse frequency substantially affects the timing, but not the total amount, of phasic LC activity. These results provide insight into VNS-evoked phasic neural activity in multiple neural structures and may be useful in guiding the selection of VNS parameters to enhance clinical efficacy.

Morphometric parameters of the VN may have a role to influence the efficacy of VNS. However, very few data are available in that area. Activation of nerve fibers during VNS depends on several factors: *i)* fibers located closer to the perimeter of the nerve and thereby closer to the VNS therapy cathode are exposed to a stronger electric field and are easier to excite than fibers located deeper in the nerve, *ii)* fibrous tissue encapsulation at the site of electrodes forms within 4–8 weeks after implantation and can increase resistance thus altering the electric field and increasing voltage requirements for fiber excitation, *iii)* fiber myelination and fiber diameter. Classically, ~20% of vagal fibers are myelinated A and B fibers and the remaining 80% are non-myelinated C-fibers; the conduction velocity of myelinated fibers is proportional to their size (Erlanger & Gasser, 1930). Vagal A-fibers are the largest and myelinated fibers and carry afferent visceral information and motor input while vagal B-fibers are small and myelinated fibers carrying parasympathetic input. Finally, vagal C-fibers are small and unmyelinated and carry afferent visceral information. C-fibers were supposed to be involved in the effects of VNS but their destruction by capsaicin did not suppressed the effects of VNS on seizures in rats (Krahl et al., 2001) thus arguing for the involvement of myelinated A and B fibers in the effect of VNS. Fibers with larger diameters require less current to reach the stimulus thresholds for recruitment and have higher conduction velocity than fibers with smaller diameters. Therefore, as current increases, fibers are recruited in the following order: A group, B group, and C group. However, successful recruitment of fibers with the same diameter varies depending upon their proximity to the stimulus source. Selective stimulation of a group of fibers to affect only a certain portion of the brain may not be effective in many patients. Achieving full activation of the VN requires selecting the right combination of VNS therapy parameter settings. Based on a computational model, Helmers et al. (Helmers et al., 2012) have shown that a range of output current settings between 0.75 and 1.75 mA with pulse width settings of 250 or 500 μs may result in optimal stimulation. The spiral electrode wrapped around the cervical left VN does not fully encircle the VN, but wraps approximately 270 degrees around it. The bipolar helical nerve electrode is the only design that is currently approved by the FDA for VNS therapy. Consequently, higher stimulation may be required for the activation of the nerve fibers present in the area not covered by the electrode. In contrast, fibers located near the perineurium of a fascicle are exposed to a stronger electric field (Helmers et al., 2012). In addition, the large variation in epineurial connective tissue might influence the effectiveness of VNS (Helmers et al., 2012). Verlinden et al. (Verlinden et al., 2016) have shown that the right cervical VN has a 1.5 times larger effective surface area than the left nerve and that there is a broad spreading within the individual nerves. They also showed that at the right side, the mean effective surface area at the cervical level is larger than at the level inside the skull base implying that the VN receives anastomosing and ‘hitchhiking’ branches from areas other than the brainstem. In addition, tyrosine hydroxylase- and dopamine ß-hydroxylase-nerve fibers have been individualized in the VN, indicating a catecholaminergic neurotransmission. Consequently, a sympathetic activation could be part of the mechanism of action of VNS. There is presently no recommendation on the positioning of the electrodes, as no method has been developed to predict whether a particular fascicle or multiple fascicles within the nerve should be recruited to elicit a therapeutic response.

All the technical points developed above should be taken into consideration in clinical studies and may influence the results of these studies.

## Conclusion

Based on the anti-inflammatory role of the VN, the use of VNS in the era of Bioelectromic Medicine opens new therapeutic avenues for the treatment of chronic inflammatory disorders such as IBD. Recent pilot studies have provided a sign in this direction. However, these data need to be confirmed in a more robust randomized control trial. In addition, looking at the optimal parameters for anti-inflammatory conditions is warranted. RA and psoriasis, other TNF mediated diseases, are also therapeutic targets of VNS.
